# Changes in verbal fluency, anthropometric parameters, physical activity, and physical fitness following an after-school basketball program in children: a pilot study

**DOI:** 10.1038/s41598-026-44652-9

**Published:** 2026-03-18

**Authors:** Christian Campos-Jara, Vanessa Carrasco-Alarcón, Sergio Araya Sierralta, Iris Paola Guzmán-Guzmán, Cristian Martinez-Salazar, Rodrigo Vargas-Vitoria, Cristián Arellano-Roco, Claudio Hernández-Mosqueira, Falonn Contreras-Osorio

**Affiliations:** 1 Exercise and Rehabilitation Sciences Institute, Faculty of Rehabilitation Sciences, Universidad Andres Bello, 7591538 Santiago, Chile; 2 Department of Physical Education, Sports, and Recreation, Pedagogy in Physical Education, School of Education and Social Sciences and Humanities, Universidad de La Frontera, 4811230 Temuco, Chile; 3 Departamento de Educación Física, Universidad de Atacama, 1531772 Copiapó, Chile; 4 Laboratory of Multidisciplinary Research and Biomedical Innovation, Faculty of Chemical-Biological Sciences, Universidad Autónoma de Guerrero, 39087 Chilpancingo de los Bravo, Mexico; 5 Faculty of Education, Universidad Católica del Maule, 3460000 Talca, Chile; 6 Departamento de Kinesiología, Universidad Católica del Maule, 3480112 Talca, Chile; 7 Departamento de Ciencias de la Educación, Universidad del Bio-Bio, 3800708 Chillán, Chile

**Keywords:** Open-skill sport, Executive function, Motor fitness, Health care, Physiology

## Abstract

Evidence suggests that open-skill team sports, such as basketball, may favor the development of executive functions in childhood. This pilot study compared pre-to-post changes in verbal fluency, anthropometric parameters, physical activity, and physical fitness between 9- to 11-year-old schoolchildren participating in a 12-week after-school basketball program and a control group. Twenty-nine participants were allocated to an intervention (basketball, *n* = 14) or control (*n* = 15) group. The intervention comprised two 60-minute sessions per week. Anthropometric parameters (height, weight, waist circumference, body mass index, and waist-to-height ratio), physical activity (PAQ-C), physical fitness (handgrip strength, standing long jump test, 10 × 5 m agility shuttle run test, and six-minute walk test), and executive functions (phonological and semantic fluency) were evaluated. The basketball group showed greater pre-to-post improvements than the control group in phonological fluency and physical fitness measures, while PAQ-C showed a trend toward between-group differences. These findings suggest that participation in a structured after-school basketball program is feasible and was associated with improvements in phonological fluency and physical fitness. However, because no active comparator was included, these changes cannot be attributed specifically to basketball; larger studies with active comparators are warranted.

## Introduction

The process of learning a sport poses a series of complex motor and cognitive challenges that drive skill acquisition^1^. This significant initial demand on cognitive processing is related to the need to develop strategies to understand skill requirements in context to achieve the desired outcome^2^. Variability then emerges as a key resource to favor cognitive development once learners perfect these strategies over time, allowing the learning curve to be maintained^3^. Open-skill sports have been described as dynamic and unpredictable environments where players are constantly challenged to achieve the game’s goal^4,5^. This involves finding relevant solutions to emerging problems arising from environment changes, for which skill and creativity are essential^6^. Basketball is an open-skill sport where players must constantly make decisions, allocating attentional resources to multiple focal points to anticipate the actions of their opponents under the pressure of time, providing an environment leading to cognitive development^7^, with special emphasis on executive functions^5,8^.

Executive functions constitute a set of voluntary mental processes that allow regulating goal-oriented behavior. They are responsible for supervising the mechanisms that mediate the use of information^9^. They relate to the ability to efficiently adapt to changing situations and analyze different variables in real time^10^. There is consensus for its classification into three main executive functions: working memory, inhibition, and cognitive flexibility^11,12^. In the educational context, there is evidence that improvements in executive functions are linked to higher academic performance in children^13,14^ and that executive functions mediate the positive relation between aerobic fitness and academic performance, especially in mathematics and language^15,16^.

Verbal fluency testing is widely used clinically and in research to assess executive functions at different developmental stages^17,18^. It is usually divided into two subtests—phonological fluency and semantic fluency—which are simple, brief, and low-cost procedures^19^. In the phonological fluency task, the person is asked to say as many words as possible beginning with a given letter (e.g., /f/, or /m/), whereas semantic fluency requires the individual to say as many words as possible corresponding to a particular category (e.g., “animals” or “fruits”) in a given time (e.g., 60 s)^20^. Several alternatives have been used as outcome measures, both in isolation and combination^21^. Among them, the number of correct words (except pronouns, synonyms, and repeated words) stands out for its ease in correction, being widely used in clinical and educational contexts^22,23^. However, the number of errors (e.g., pseudo-words or repetitive words)^23,24^, as well as switching and clustering measures (e.g., number of switches, number of clusters, and the mean cluster size) have been mostly used in the research setting to analyze the processes underlying these tasks^22,25^.

Regarding the contribution of executive functions to performance in verbal fluency tasks, working memory stands out as a predictor of performance in both subtests^26^, although the contribution of planning skills (a high-level executive function)^26^, cognitive flexibility^25^, and inhibitory control^27^ is also identified. However, phonological and semantic fluency performance could reflect the involvement of different cognitive resources depending on the specific requirements of each task^27^. As the generation of words based on phonemic criteria is unusual, it has been described that the phonological fluency task requires greater effort to search and organize language, making it necessary to suppress the usual trend to organize words based on their meaning and semantic associations^28^, depending to a greater extent on frontal lobe maturation^29^ and processes linked to inhibitory control^27^. Moreover, grouping and switching strategies would be useful when applied together to optimize performance in both verbal fluency tasks^30^ as they are especially relevant for word retrieval in the phonological fluency task^27^.

Although there is ample evidence linking sport participation to cognitive benefits related to executive functions, specifically within the open- versus closed-skill framework^5^, basketball intervention studies in children remain comparatively limited and heterogeneous in both training formats and cognitive outcomes. Nevertheless, available basketball-based interventions in school-aged children suggest improvements in executive-function outcomes, including gains in working memory, inhibitory control and cognitive flexibility, as well as broader composite measures of executive functioning^31–33^. Notably, in boys aged 6–8 years, higher basketball training frequency (≥ 2 sessions/week) was associated with better working memory performance (higher N-back accuracy) and more efficient cognitive flexibility (higher switching-task accuracy and shorter reaction time), whereas inhibitory control did not differ across training-frequency groups^33^. Recent meta-analyses indicate that open-skill exercise interventions are associated with improvements in executive functions in children and provide quantitative support for the open- versus closed-skill framework relevant to basketball as a strategic open-skill sport^34,35^.

Building on this evidence, verbal fluency tasks offer a meaningful and accessible way to examine cognitive engagement in children, as they are widely recognized to involve core executive control processes—such as inhibition, working memory updating and cognitive flexibility^25–27^—while also reflecting a distinct linguistic ability relevant to academic and communicative development^16,29^. Therefore, in the present study, verbal fluency was selected as a cognitive domain that bridges executive and language functions, allowing for the exploration of specific cognitive changes potentially associated with basketball practice without assuming generalized effects across all executive domains.

Beyond cognitive outcomes, basketball training programs in children have also been associated with improvements in physical activity and key components of physical fitness such as speed/agility and muscular strength/power^36–38^, while anthropometric indicators (e.g., body mass index and waist-to-height ratio) provide widely used health-related markers of physical adaptation to sport participation. For example, mini-basketball interventions have reported improvements in speed/agility and muscular strength in children (e.g., shuttle-run and standing long jump performance)^36^. In addition to these gross-motor components, basketball participation has been associated with better fine motor/neuromotor skills such as manual dexterity in youth, which integrates cognitive processing and precise force control^39^. Although manual dexterity was not assessed in the present study, this evidence supports a broader view of the motor skills potentially influenced by basketball in childhood. Because physical fitness and habitual activity can be functionally related to cognitive performance in school-aged children^15,16,40,41^, jointly assessing verbal fluency, physical activity, physical fitness and anthropometric parameters offers a more integrated perspective on how basketball practice may contribute to both cognitive and physical development in childhood.

Therefore, this study aims to analyze the effects of basketball practice on verbal fluency, anthropometric parameters, physical activity and physical fitness in children who face the process of learning this sport in an after-school context for the first time, compared to a control group of students who do not perform physical activity in addition to their regular classes.

## Methods

### Ethical clearance

The study protocol was approved by the scientific ethics committee of Universidad Andrés Bello, Chile (No. 009/2021). The procedures were performed as per the Declaration of Helsinki (2013). Written informed consent was obtained from the parents or legal guardians of all participating children prior to their inclusion in the study. In addition, all children provided written informed assent after receiving an age-appropriate explanation of the study procedures.

### Participants

The study included boys (*n* = 16) and girls (*n* = 13) between 9 and 11 years old (mean ± standard deviation = 10.68 ± 0.55 years) of a medium socioeconomic level^42^ who attended a public educational establishment in the city of Rancagua, Chile. Students were classified into two groups according to their decision to participate in an after-school sports workshop: (1) Control group: boys and girls who did not take part in sports activities in addition to their physical education classes; (2) Boys and girls who engaged in an after-school basketball workshop. Because enrollment in the after-school basketball workshop was voluntary, group allocation reflected self-selection rather than random assignment, which may be associated with unmeasured differences (e.g., motivation, parental support, or baseline activity preferences). In the informed consent, their tutors provided information regarding the following exclusion criteria: (a) medical history that would prevent sports practice (e.g., severe visual impairment or osteoartromuscular injury), (b) a disorder affecting neurodevelopment (e.g., attention deficit hyperactivity disorder), (c) regular practice of physical exercise or sport at least once a week outside the school context, and (d) previous basketball experience. The latter criterion follows that of previous studies that have shown that sports experience can impact cognitive outcomes, especially those related to prefrontal cortex functioning^43^.

### Study design

A quasi-experimental pilot study with pre- and post-test measures was conducted to compare pre-to-post changes in verbal fluency, anthropometric parameters, physical activity, and physical fitness between an after-school basketball group and a control group. The intervention period lasted 12 weeks, with two hours of weekly training. The control group continued with their usual physical activity routine and did not participate in an additional structured after-school program (i.e., no active comparator).

### Measures

#### Verbal fluency

The “Verbal Fluency” test of the Neuropsychological Assessment for Executive Functions in Children (ENFEN) battery was used^44^. It comprises two parts: phonological fluency and semantic fluency. Application is individual, always performed in the above-mentioned order and both include training rehearsal. In phonological fluency, the child was asked to say as many words as possible beginning with the letter “M” in one minute, whereas in semantic fluency, the task was to list as many “animals” as possible in one minute. The number of correct words, excluding repetitions, derivatives, and superordinates, was recorded as a direct score^25,45^.

#### Physical activity

Participants’ physical activity was measured through the Physical Activity Questionnaire for older Children (PAQ-C), Spanish version^46^. This questionnaire provides a measure of the physical activity performed in the last seven days through nine questions asked in a direct interview format. Responses are rated on a 5-point scale.

#### Anthropometric parameters

Both height and body weight were assessed using the procedure described by Ross and Marfell-Jones^47^. During the assessment, the children were asked to wear a T-shirt, shorts, and socks, without shoes. A portable stadiometer (Seca & Co., KG, Hamburg, Germany) and a digital scale (TANITA^™^, model 331, Tokyo, Japan) were used, and once both measurements were obtained, the body mass index (BMI) was calculated by dividing body mass by height squared (kg/m^2^). Waist circumference (WC) was measured with a flexible tape measure, and the waist-to-height ratio (WtHR) was obtained by dividing WC by height^48^.

#### Physical fitness

The standing long jump test (SLJ)^49^ was used to assess lower-body muscular power. Children were asked to jump forward as far as possible, swinging their arms, bending their knees, and simultaneously lifting both feet off the ground. The distance in centimeters (cm) from the starting line to the farthest point of contact by the heels when landing was recorded. The 10 × 5 m agility shuttle run test^49^ was used to assess sprint performance, during which participants had to run as fast as possible between two cones separated by 5 m, until reaching a total distance of 50 m (5 round trips). The total time spent on the task (seconds) was recorded. Handgrip strength (HGS) was used as a measure of upper-body muscular strength (kg) using a hydraulic dynamometer (JAMAR Hydraulic Hand Dynamometer^®^ Model PC-5030 J1, Fred Sammons, Inc., Burr Ridge, IL, USA). To this end, the children were asked to hold the dynamometer with the dominant hand while extending their arm to the side of their bodies and exerting the maximum possible grip force for at least 2 s. The best value from a total of two attempts was considered^49^. Finally, a 6-minute walk test (SMWT) was used to assess aerobic fitness^50^. Participants were asked to walk as fast as possible in a 30-meter corridor for a total of 6 min, recording the total distance traveled in meters.

#### Procedure

In the first instance, an informative meeting was organized at the school to provide in-depth information to both parents and interested children. Written informed consent and assent forms were then handed out for review and signature. Participants were informed that the basketball workshop would have a total capacity of 16 participants due to infrastructure limitations and that the control group would have the option of holding the workshop in a second implementation stage, during the following semester. As this study was designed as a quasi-experimental pilot in an authentic school setting, a formal a priori sample size/power calculation was not performed. The target sample was therefore determined pragmatically by feasibility, primarily constrained by the maximum workshop capacity (16 places) and participant availability/consent. The pre-intervention assessment was conducted one week before the start of the workshop, and tests were performed in counterbalanced order, divided into three stages: (1) Verbal fluency and PAQ-C; (2) Anthropometric parameters; (c) Physical fitness. Each child was assigned an alphanumeric code to ensure data anonymization. Evaluators were unaware of the identity of participants and their assignment to groups. The intervention period lasted for a total of 12 weeks, with two weekly training sessions of 1 h each. The basketball training was guided by a physical education teacher with more than 15 years of experience in teaching the sport to children, who recorded attendance and adverse events in every session. She also asked children to assess their level of perceived effort using the EPInfant scale^51^ at the end of each training session. This scale has visual support (illustrations), along with numerical and verbal descriptors (written sentences) for each level of effort to facilitate children’s understanding. The control group was told not to make any changes in their physical activity routine during the intervention period, which was confirmed by a member of the research team by means of a telephone call to each tutor every 2 weeks. Once the intervention phase was over, assessments were applied again to both groups, following the same procedure described above.

All physical activity, anthropometric, and physical fitness assessments were conducted by a single evaluator with a university degree in Physical Education and over five years of experience administering motor tests in children. Verbal fluency assessments were performed by a speech-language pathologist with more than five years of experience in school-age language evaluation. Prior to data collection, both assessors participated in an induction and calibration session to standardize administration and recording procedures according to the technical guidelines previously established for each assessment protocol. All assessments were supervised by the study coordinator to ensure procedural consistency and compliance with the established protocols.

#### Basketball group

The children who participated in the sports intervention performed an additional 120 min of physical activity per week, distributed in two sessions of equal duration. The workshop took place after the school day, on days other than those when they had their regular physical education classes at school. The basketball training method incorporated sets of tasks, exercises, or games combining the phases of attack, defensive balance, defense, and counterattack, either analytically or in combination. Moreover, variables such as game space, number of players, and resolution time were manipulated. The program structure comprised 10 min of basic sports training, 40 min of the main exercise, and 10 min of cool down with stretching. Basic sports training included progressive movements, joint mobility and partner work (e.g., jumping over a partner). The main exercise entailed 5 min of individual improvement tasks (e.g., 1 × 1 throwing), 10 min of one-phase improvement tasks (e.g., 3 × 2 in three zones of the attacking field), and 10 min of phase combination tasks (e.g., 3 × 3 defensive counterattack-balance), starting with a time-limited defensive phase to solve the task, and 15 min for the actual game (e.g., matches with situations of equality and numerical inequality), seeking variability in the task^3^. The last 10 min of each session were dedicated to the cool-down routine, which included stretching exercises and a reflective group discussion guided by the teacher. During this phase, children rated their perceived level of effort using the EPInfant scale^51^. Although not analyzed as an outcome variable, this measure formed part of the metacognitive component of the intervention, promoting self-awareness of physical engagement and emotional responses. In addition, the perceived effort reports provided qualitative feedback to the instructor, allowing for adjustments in session intensity and supporting individualized regulation of task demands.

### Statistics and analysis

Descriptive statistics were used to summarize the sample. Categorical variables are presented as frequencies and percentages, and continuous variables as means ± standard deviation (SD). Baseline between-group differences were examined using independent-samples t-tests for continuous variables and χ^2^ tests for categorical variables. Within-group pre-to-post changes were examined using paired t-tests. No a priori sample size/power calculation was performed because this study was designed as a feasibility-oriented quasi-experimental pilot. As a quantitative indication of statistical power for the primary outcome (phonological fluency), we conducted a post hoc sensitivity analysis for the ANCOVA comparison (α = 0.05, two-sided). With a total sample size of *N* = 29 (df = 1, 26), the design had approximately 80% power to detect large effects of about partial η^2^_*p*_ ≈ 0.23 or greater, indicating that smaller effects may have been underpowered in this feasibility-oriented pilot. The primary feasibility-oriented analysis compared individual pre-to-post change scores (delta; Δ) between groups using independent-samples t-tests. In addition, to account for baseline imbalances, we conducted ANCOVA sensitivity analyses for each outcome, using the post-intervention value as the dependent variable, group as the fixed factor, and the corresponding baseline value as a covariate; ANCOVA results are reported as F-statistics, P-values, and partial η^2^_*p*_ (Table 2). Values of η^2^_*p*_ were interpreted as small (< 0.06), moderate (0.06–0.14), and large (≥ 0.14). Pearson’s correlation coefficient (r) was used to quantify associations between change scores within groups. The criteria to interpret the magnitude of the r were null (0.00–0.09), small (0.10–0.29), moderate (0.30–0.49), large (0.50–0.69), very large (0.70–0.89), nearly perfect (0.90–0.99), and perfect (1.00). A binary logistic regression was performed to examine whether the change in standing long jump predicted the likelihood of improvement in phonological fluency (improved vs. not improved). Odds ratios (OR) with 95% confidence intervals were reported. Exploratory comparisons by sex were conducted for baseline and post-intervention verbal fluency scores using independent-sample t-tests to identify potential differences between boys and girls. All data were analyzed using STATA v16.0 software (StataCorp LLC, College Station, TX, USA). Statistical significance was set at *P* ≤ 0.05.

## Results

### Characteristics of the study sample

The final sample comprised 29 young children (13 females and 16 males) aged 110–132 months, around 9–11 years (Mean = 10.68, SD = 0.55), divided in a control group and an experimental group (basketball intervention). As shown by analysis of Student’s *t* test and chi-square test, groups did not significantly differ regarding age, physical activity score related to PAQ-C and 10 × 5 m agility shuttle run test (s), and phonological and semantic fluency. However, the basketball group shows higher anthropometric parameters as well as better handgrip strength and score for SMWT and SLJ tests. Details are provided in Table 1.

No significant differences were observed between boys and girls in baseline phonological or semantic fluency scores (*p* > 0.05). Similarly, no post-intervention differences were found between sexes for either measure (*p* > 0.05). These exploratory comparisons indicate that sex did not influence verbal fluency performance in this pilot sample.

**Table 1:** Anthropometric, physical, and executive function components in participants.

Characteristics	All (*n* = 29)	Control group (*n* = 15)	Basketball group (*n* = 14)	*p*-value
Age (yrs)	10.68 ± 0.55	10.68 ± 0.26	10.67 ± 0.76	0.96
Sex n (%)				0.83
Girls	13 (44.83)	7 (46.67)	6 (42.86)	
Boys	16 (57.14)	8 (53.33)	8 (57.14)	
Anthropometrics				
Weight (kg)	42.25 ± 8.89	38.2 ± 3.78	46.58 ± 10.76	0.008
Height (m)	1.46 ± 0.07	1.47 ± 0.06	1.46 ± 0.08	0.66
WC (cm)	69.88 ± 9.19	65.13 ± 3.31	74.97 ± 10.79	0.002
WtHR (WC/height)	0.47 ± 0.06	0.44 ± 0.02	0.51 ± 0.07	0.001
BMI (kg/m^2^)	19.49 ± 3.56	17.55 ± 1.31	21.56 ± 4.05	0.001
Physical activity				
PAQ-C	3.4 ± 0.64	3.57 ± 0.21	3.24 ± 0.85	0.168
Physical fitness				
SMWT (m)	482.8 ± 128.7	401.2 ± 127.8	570.28 ± 46.6	0.001
10 × 5 m sprint (s)	21.99 ± 1.69	21.98 ± 0.64	22.01 ± 2.39	0.968
SLJ (cm)	130.41 ± 17.03	138.6 ± 15.7	121.64 ± 14.18	0.005
HGS (kg)	14.82 ± 2.5	15.53 ± 1.68	16.21 ± 2.54	0.002
ENFEN tasks				
Phonological fluency (score)	10.62 ± 2.16	10.6 ± 2.13	10.64 ± 2.27	0.958
Semantic fluency (score)	12.03 ± 2.17	12.0 ± 1.69	12.07 ± 2.67	0.931

The quantitative data shown represents the mean ± SD. Values between groups were analyzed using the Student´s -*t* test. Qualitative data are presented as proportions (percentages) and analyzed using the Chi-square test. *P* < 0.05 was considered statistically significant. ENFEN = Neuropsychological Assessment for Executive Function in Children Battery; BMI = body mass index; WC = waist circumference; WtHR = waist-to-height ratio; PAQ-C = Physical Activity Questionnaire for Older Children; SMWT = 6-minute walk test; SLJ = standing long jump test; HGS = handgrip strength.

### Between-group differences in verbal fluency, anthropometric, and physical outcomes

**Fig. 1:**
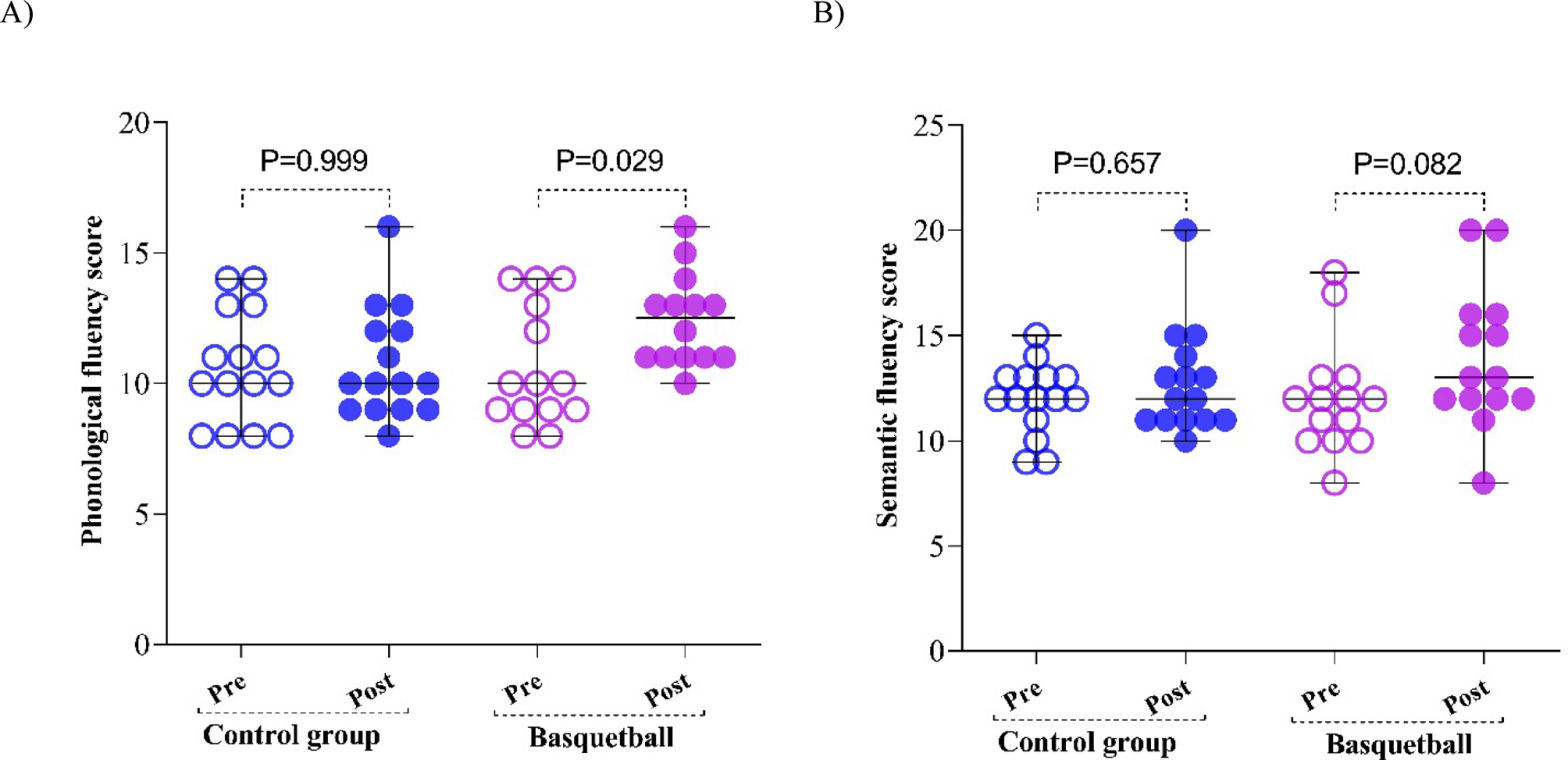
Phonological (**A**) and semantic (**B**) verbal fluency scores at pre- and post-assessment in the basketball and control groups. Statistical analyses were performed using Student’s t-test; *p* < 0.05 was considered statistically significant.

Figure 1 shows pre-to-post changes in semantic (*p* = 0.082) and phonological (*p* = 0.029) fluency in the basketball and control groups. Between-group differences in change scores were tested using independent-samples t-test.

Table 2 summarizes ANCOVA sensitivity analyses in which post-intervention outcomes were compared between groups adjusting for the corresponding baseline values. Overall, no evidence of between-group differences was observed for anthropometric variables (all *p* > 0.05). For physical activity, PAQ-C showed a non-significant trend toward a between-group difference (*p* = 0.080; partial η^2^_*p*_ = 0.113). Of note, PAQ-C showed a significant between-group difference in the change-score analysis (Table 3), whereas the baseline-adjusted ANCOVA yielded a non-significant trend, suggesting that this finding may be sensitive to analytical approach and baseline variability. In contrast, the basketball group showed evidence of superior post-intervention physical fitness performance after baseline adjustment, with large effects for the 6-minute walk test (SMWT; *p* < 0.001; partial η^2^_*p*_ = 0.625), the 10 × 5 m agility shuttle run test (*p* < 0.001; partial η^2^_*p*_ = 0.668), standing long jump (SLJ; *p* < 0.001; partial η^2^_*p*_ = 0.447), and handgrip strength (HGS; *P* < 0.001; partial η^2^_*p*_ = 0.493). Regarding executive function, ANCOVA indicated a significant between-group difference in phonological fluency, favoring the basketball group (*p* = 0.003; partial η^2^_*p*_ = 0.286), whereas semantic fluency did not differ between groups (*p* = 0.247; partial η^2^_*p*_ = 0.051).

Table 3 describes the pre-to-post change scores in the basketball group, as well as the pre-to-post change scores in the control group. Both study groups showed negative deltas of change for anthropometric parameters, which refers to a decrease in weight, BMI, or waist-to-height ratio. However, this change was not statistically significant. With respect to executive function, the basketball group showed an improvement in their phonological fluency (*p* = 0.027). Moreover, when comparing change scores between groups, it was found that the basketball group showed significantly greater improvements for the physical function variables PAQ-C (*p* = 0.042), SMWT, SLJ, HGS, 10 × 5 m sprint (*p* < 0.001), as well as for the executive function of phonological fluency (*p* = 0.009).

**Table 2:** Pre- and post-intervention outcomes and baseline-adjusted ANCOVA results by group.

Characteristics	*n*	Control group		*n*	Basketball group		F	*P*-value	Partial η^2^_*p*_
		Pre-test Mean (SD)	Post-test Mean (SD)		Pre-test Mean (SD)	Post -test Mean (SD)			
Anthropometrics									
Weight (kg)	15	38.2 ± 3.78	38.6 ± 4.73	14	46.58 ± 10.76	46.15 ± 10.49	2.734	0.110	0.095
Height (m)	15	1.46 ± 0.07	1.48 ± 0.05	14	1.46 ± 0.08	1.47 ± 0.08	1.219	0.280	0.045
WC (cm)	15	69.88 ± 9.19	64.6 ± 3.77	14	74.97 ± 10.79	74.47 ± 10.74	0.021	0.887	0.001
WtHR (WC/height)	15	0.47 ± 0.06	0.43 ± 0.02	14	0.51 ± 0.07	0.50 ± 0.07	0.761	0.391	0.028
BMI (kg/m^2^)	15	19.49 ± 3.56	17.45 ± 1.38	14	21.56 ± 4.05	21.2 ± 3.83	0.003	0.958	0.000
Physical activity									
PAQ-C	15	3.57 ± 0.21	3.65 ± 0.23	14	3.24 ± 0.85	3.62 ± 0.92	3.318	0.080	0.113
Physical fitness									
SMWT (m)	15	482.8 ± 128.7	400.4 ± 123.2	14	570.28 ± 46.6	582.8 ± 41.4	43.246	˂0.001	0.625
10 × 5 m sprint (s)	15	21.99 ± 1.69	22.19 ± 0.81	14	22.01 ± 2.39	20.86 ± 2.11	52.321	˂0.001	0.668
SLJ (cm)	15	130.41 ± 7.03	137.9 ± 4.33	14	121.64 ± 4.18	127.5 ± 15.41	21.028	˂0.001	0.447
HGS (kg)	15	14.82 ± 2.5	13.73 ± 1.75	14	16.21 ± 2.54	17.14 ± 2.14	25.313	˂0.001	0.493
ENFEN tasks									
Phonological fluency (score)	15	10.62 ± 2.16	10.73 ± 2.12	14	10.64 ± 2.27	12.42 ± 1.74	10.420	0.003	0.286
Semantic fluency (score)	15	12.03 ± 2.17	12.8 ± 2.51	14	12.07 ± 2.67	13.92 ± 3.33	1.400	0.247	0.051

Data are presented as mean ± SD. *P* < 0.05 was considered statistically significant. ANCOVA results are reported as F-statistics, P-values, and partial eta squared (η^2^_*p*_). ENFEN = Neuropsychological Assessment for Executive Function in Children Battery; BMI = body mass index; WC = waist circumference; WtHR = waist-to-height ratio; PAQ-C = Physical Activity Questionnaire for Older Children; SMWT = 6-minute walk test; SLJ = standing long jump test; HGS = handgrip strength

**Table 3:** Anthropometric, physical, and executive function components in the participants divided by participant groups.

Characteristics	Control group		Pre-Post delta; *p*-value	Basketball group		Pre-Post delta; *p*-value	*P*-value between ∆ values
	Pre	Post		Pre	Post		
Anthropometrics							
Weight (kg)	38.2 ± 3.78	38.6 ± 4.73	∆ = 0.39; ns	46.58 ± 10.76	46.15 ± 10.49	∆ = −0.46; ns	0.067
Height (m)	1.46 ± 0.07	1.48 ± 0.05	∆ = 0.01; ns	1.46 ± 0.08	1.47 ± 0.08	∆ = 0.006; ns	0.287
WC (cm)	69.88 ± 9.19	64.6 ± 3.77	∆ = −0.46; ns	74.97 ± 10.79	74.47 ± 10.74	∆ = −0.50; ns	0.911
WtHR (WC/height)	0.47 ± 0.06	0.43 ± 0.02	∆ = −0.006; ns	0.51 ± 0.07	0.50 ± 0.07	∆ = −0.005; ns	0.737
BMI (kg/m^2^)	19.49 ± 3.56	17.45 ± 1.38	∆ = −0.09: ns	21.56 ± 4.05	21.2 ± 3.83	∆ = −0.35; ns	0.239
Physical activity							
PAQ-C	3.57 ± 0.21	3.65 ± 0.23	∆ = 0.08; ns	3.24 ± 0.85	3.62 ± 0.92	∆ = 0.38; ns	0.042
Physical fitness							
SMWT (m)	482.8 ± 128.7	400.4 ± 123.2	∆ = −0.80; ns	570.28 ± 46.6	582.8 ± 41.4	∆ = 12.57; ns	0.001
10 × 5 m sprint (s)	21.99 ± 1.69	22.19 ± 0.81	∆ = 0.20: ns	22.01 ± 2.39	20.86 ± 2.11	∆ = −1.14; ns	0.001
SLJ (cm)	130.41 ± 7.03	137.9 ± 4.33	∆ = −0.66: ns	121.64 ± 4.18	127.5 ± 15.41	∆ = 5.85; ns	0.001
HGS (kg)	14.82 ± 2.5	13.73 ± 1.75	∆ = 0.20; ns	16.21 ± 2.54	17.14 ± 2.14	∆ = 0.92; ns	0.001
ENFEN tasks							
Phonological fluency (score)	10.62 ± 2.16	10.73 ± 2.12	∆ = 0.13; ns	10.64 ± 2.27	12.42 ± 1.74	∆ = 1.78; 0.027	0.009
Semantic fluency (score)	12.03 ± 2.17	12.8 ± 2.51	∆ = 0.80; ns	12.07 ± 2.67	13.92 ± 3.33	∆ = 1.85; ns	0.255

The data shown represents the mean ± SD. Between-group differences in change scores (Δ = post–pre) were tested using Student’s t-test; *P* < 0.05 was considered statistically significant (ns = not significant). ENFEN = Neuropsychological Assessment for Executive Function in Children Battery; BMI = body mass index; WC = waist circumference; WtHR = waist-to-height ratio; PAQ-C = Physical Activity Questionnaire for Older Children; SMWT = 6-minute walk test; SLJ = standing long jump test; HGS = handgrip strength. Statistical significance was set at *P* < 0.05 for between-group differences.

### Effects of the basketball intervention on improving phonological fluency

**Fig. 2:**
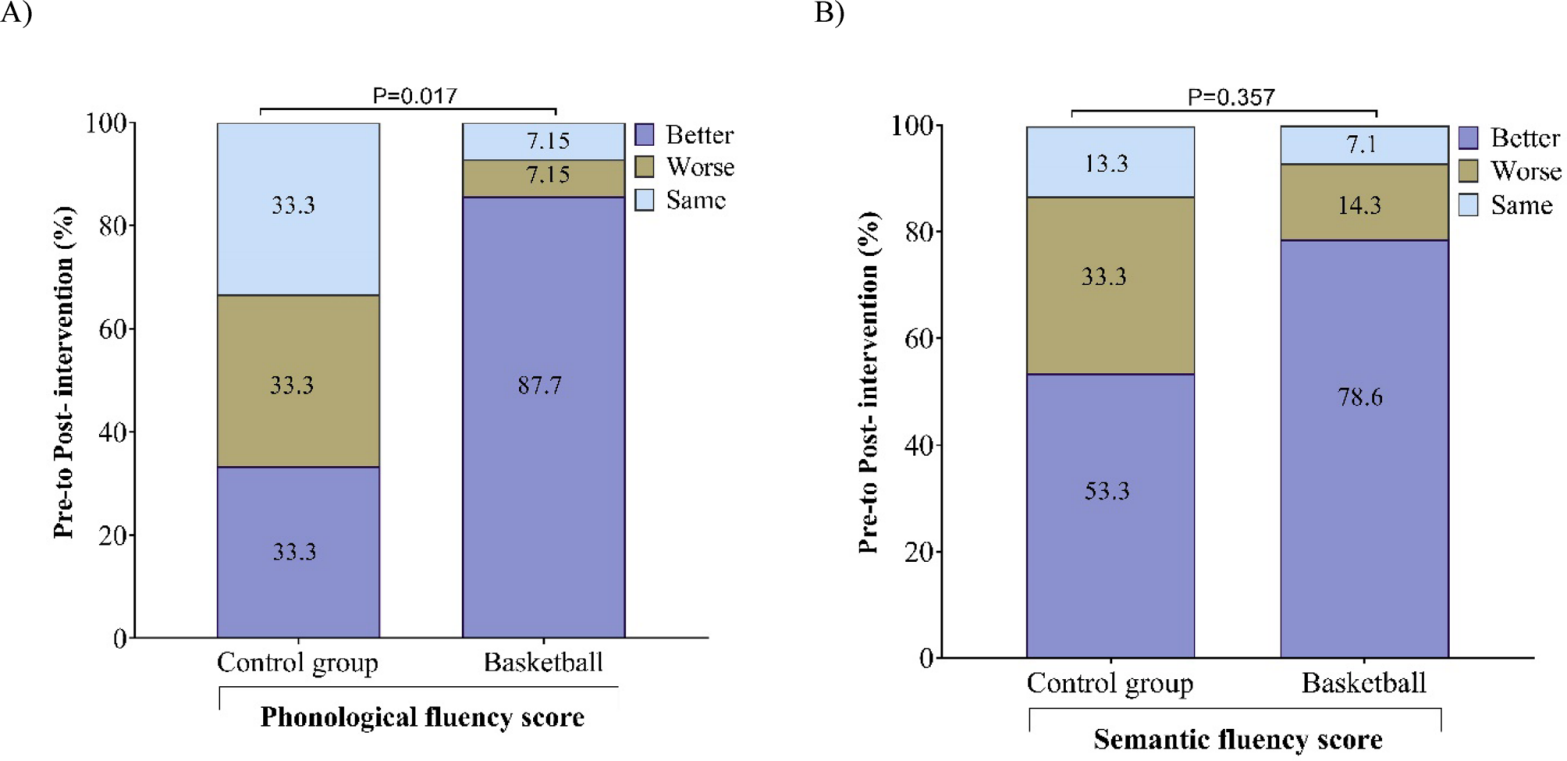
Proportion of children with change between Pre-Post evaluation for phonological fluency (**A**) and semantic fluency (**B**) according to participant groups.

The change in the delta of semantic and phonological fluency scores was determined for each participant, categorizing positive delta as “better” and negative changes as “worse.” Values with no change or null values were the “same.” Fig. 2 shows that for the semantic fluency score, 7.1% of the children remain with the same semantic fluency post basketball intervention, compared to the children in the control group, where the proportion is nearly the double (13.3%). Similarly, 14.3% and 33.3% of the participants had worsened scores in the intervention and control groups, respectively. Moreover, the difference in the proportion of participants who improved in this task was greater by 25.3% in the basketball group compared to the control group. The difference between groups was even more evident and statistically significant for the phonological fluency task, where the difference in the improvement proportion reached 54.4% in favor of the intervention group, with respect to that found in the control group. In this task, only 7.15% of the children maintained worse or equal scores in the basketball group (Fig. 2).

**Fig. 3:**
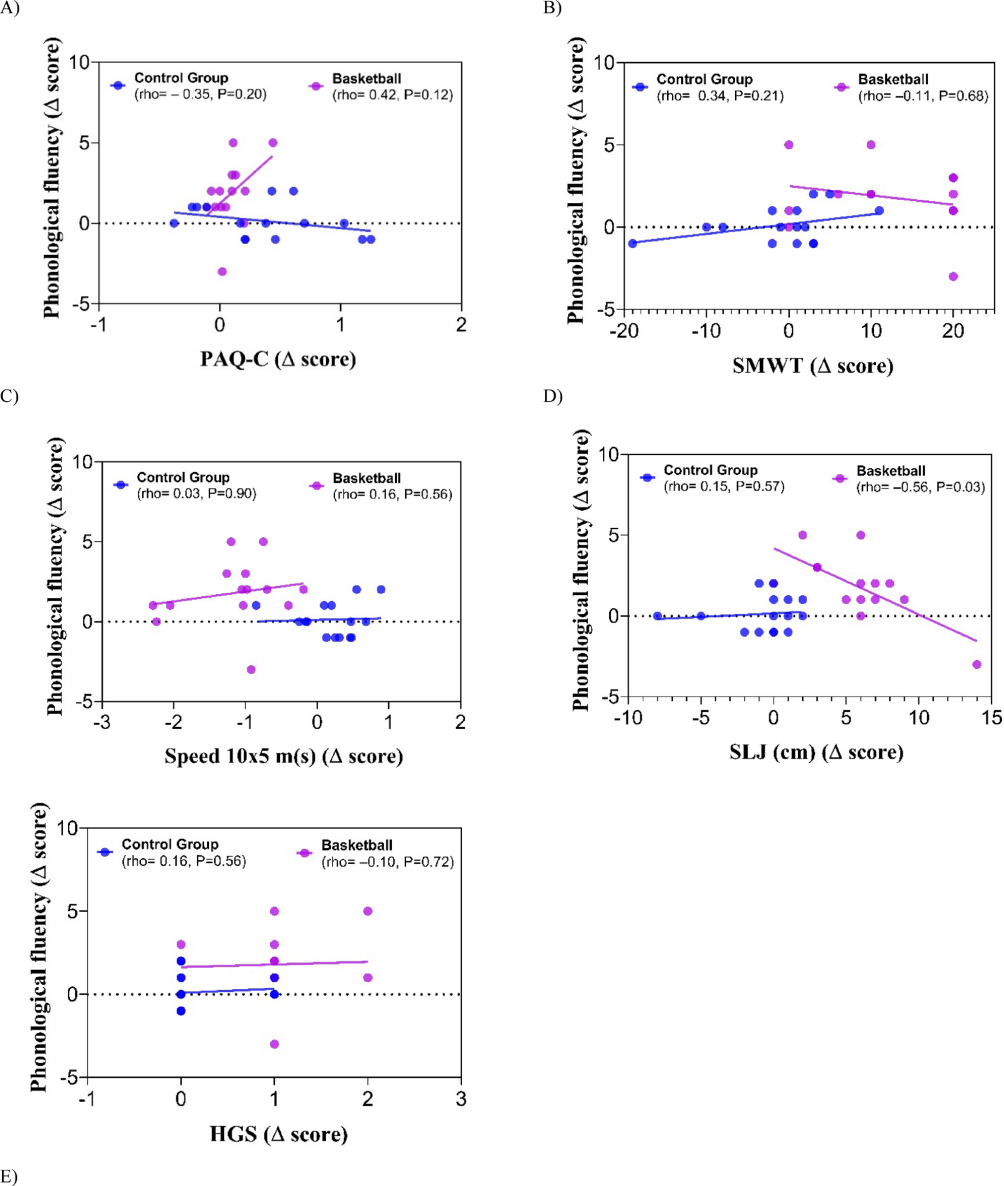
Correlation of Phonological Fluency score for Pre-Post change evaluation with Pre-Post change on physical parameters such as PAQ-C (**A**), SMWT (**B**), Speed 10 × 5 m (**C**), SLJ (**D**), and HGS (**E**) according to groups.

Furthermore, the linear relationship between the deltas of change of phonological fluency and that of the parameters that analyze physical fitness was evaluated, observing a moderate positive linear relationship between phonological fluency and PAQ-C (*r* = 0.42, *p* = 0.12) in the basketball intervention group. In the control group, this relation was negative (*r* = − 0.35, *p* = 0.20). The relationship between phonological fluency was significantly related to change in long jump (*r* = − 0.56, *p* = 0.03) (Fig. 3), which in a logistic regression analysis showed an association of OR = 4.55 (0.91–22.6), *p* = 0.064, with improvement in phonological fluency.

### Adverse events

No adverse events deriving from the implementation of the program were identified.

## Discussion

This study analyzed the effects of a pilot after-school basketball intervention on verbal fluency, anthropometric parameters, physical activity and physical fitness of 9 to 11-year-old school-aged children after a 12-week intervention period, compared to a control group that continued with their usual school routine. Sports practice incorporated manipulation of variables such as game area, number of players and resolution time to add variability to training, contributing to increased cognitive demand^3^. The intervention group experienced improvements in phonological fluency compared to the control group. Further, when comparing change scores (Δ) between groups, the basketball training group showed greater improvements in phonological fluency and in physical fitness outcomes, and higher PAQ-C change scores. In baseline-adjusted ANCOVA sensitivity analyses (Table 2), between-group differences remained evident for physical fitness outcomes and phonological fluency, whereas PAQ-C showed only a non-significant trend. These baseline imbalances are consistent with the voluntary nature of workshop enrollment and suggest that pre-existing characteristics (e.g., higher fitness levels or family support) may have contributed to group differences observed at follow-up. However, given the quasi-experimental pilot design, voluntary enrollment, and the absence of an active comparator, these between-group differences should be interpreted as associations rather than basketball-specific effects. In addition, the linear relationship between the deltas of change in phonological fluency and physical activity level showed a moderate positive association in the intervention group. The control group did not experience significant improvements in the variables evaluated.

### Executive function: phonological fluency

Phonological fluency was assessed using a subtest of the Neuropsychological Assessment for Executive Functions in Children (ENFEN) battery^44^. Our analyses revealed a significant improvement in the group participating in the basketball sports intervention compared to the control group, which did not experience significant changes. This between-group difference was also supported by baseline-adjusted ANCOVA results (Table 2). Studies agree with these findings as they prove that basketball increases both general measures of executive functions^31^ and specific measures in the dimensions of working memory and inhibition^32^, key elements for the lexical selection required in phonological fluency tasks^26,27^. Moreover, Xu et al.^33^ support the benefits of regular basketball practice on working memory and cognitive flexibility in children, showing better results as a function of training frequency (2 or more sessions per week).

Taken together, the basketball literature in children remains heterogeneous in both intervention format and cognitive endpoints: prior studies have reported improvements in general or domain-specific executive-function outcomes (e.g., working memory, inhibition, and cognitive flexibility), but the measures used are not always directly comparable to verbal fluency tasks. Within this context, our finding of improved phonological fluency can be interpreted as consistent with the broader pattern of executive-control benefits reported in pediatric basketball research, while remaining cautious about cross-task generalization^31–33^.

The improvement observed in phonological fluency should be interpreted as a domain-specific cognitive enhancement rather than a generalized effect on executive functions^17,18^. Verbal fluency is a complex linguistic task that draws upon executive processes—such as inhibition of competing responses, strategic retrieval, and monitoring^27^—but it also reflects broader language-related abilities that are essential for academic achievement and social communication^16,29^. This dual nature makes verbal fluency a particularly valuable outcome in childhood, as it bridges cognitive control and linguistic competence within natural learning contexts, such as sport-based activities.

Previous systematic reviews, such as those carried out by Contreras-Osorio et al.^8^, Song et al.^52^ and Mao et al.^53^ describe that cognitively engaging physical activity interventions, including sport-based interventions, have a positive impact on the executive functions of children and adolescents in their three main dimensions (working memory, cognitive flexibility, and inhibition). Based on such improvements, different mechanisms have been described, such as increased cerebral blood flow^54^ and synergistic activation of neural networks linked to both cognition and movement, driven by demanding, novel, unpredictable and rapid environmental stimuli related to activities involving social, motor and cognitive demands in an integrated manner^1,55^. It has also been proposed that increased brain-derived neurotrophic factor (BDNF) release from cognitively demanding physical activity may play a role in cognitive enhancement^56,57^. Because we did not directly assess physiological mediators (e.g., cerebral blood flow or neurotrophic factors) or psychological mediators (e.g., motivation, enjoyment, or perceived competence), these proposed pathways should be considered plausible mechanisms rather than demonstrated explanations in the present pilot study.

No sex-related differences were observed in phonological or semantic fluency, suggesting that the observed improvements were primarily driven by participation in the basketball program rather than baseline gender-related linguistic advantages. Although some evidence indicates a slight female advantage in verbal fluency, the considerable variability reported across studies suggests that this difference is strongly influenced by factors such as language, cultural context, and country of origin^58^.

### Physical activity and executive function

Physical activity was assessed using the Physical Activity Questionnaire for older Children (PAQ-C), Spanish version^46^. This is a self-reported measure of physical activity over the previous 7 days, with higher scores indicating higher activity levels. In the change-score (Δ) analysis, children in the basketball group showed greater increases in PAQ-C than controls, and PAQ-C change was positively related to phonological fluency. However, this finding should be interpreted cautiously because PAQ-C showed only a non-significant trend in the baseline-adjusted ANCOVA sensitivity analysis, suggesting that the result may be sensitive to analytical approach and baseline variability. These observations in physical activity levels and the relationship between this increase and higher performance in executive functions in sports intervention programs by schoolchildren are supported by previous studies conducted in children^39,59–61^. Contreras et al. (2022)^40^ analyzed the effects of two sports programs (handball and athletics) implemented in after-school hours with a school population (10–12 years old), showing that increases equal to or greater than 0.5 points in PAQ-C were associated with significant increases in cognitive flexibility, working memory, attention, and planning after the 12-week intervention. However, Zeng et al.^59^ analyzed the association between daily physical activity and after-school sedentary time with executive function in children aged between 6 and 12 years old, classifying participants into 4 groups. Their results showed that the group considered as low after-school sedentary time/high physical activity showed a positive association with executive function. Padmapriya et al. (2024)^60^ conducted a prospective analysis between specific domains of physical activity and executive functions, showing that children who participated in organized sports at 5.5 and 8 years old had higher executive function at 8.5 years old. These findings are also supported by Koepp and Gershoff (2022)^61^, who reported that increased physical activity resulting from participation in group sports predicts improvements in executive functions for the dimensions of inhibitory control, attention, and social self-control in both kindergarten to 1st grade and 3rd to 4th grade children. These results could be related to the process of developing functional motor skills in enriched environments, fostering challenging complex and participatory motor experiences driving cognitive development in terms of executive functions^41^.

In line with this interpretation, basketball may represent a particularly cognitively engaging form of organized physical activity because it requires continuous monitoring of dynamic play situations, rapid decision-making, and flexible adaptation of motor responses to changing constraints^33^. These task demands align with core executive-control processes (e.g., inhibition, working memory updating, and cognitive flexibility), providing a plausible—although not basketball-specific—pathway through which structured participation could be associated with the improvements observed in this pilot study^52^.

### Physical fitness and executive function

Physical fitness was evaluated using the standing long jump^48^, 10 × 5 m agility shuttle run^49^, handgrip strength^49^ and 6-minute walk tests^50^ to assess lower-body muscular power, sprint performance, upper-body muscular strength, and aerobic fitness, respectively. When comparing the deltas of change between study groups, our results found that children who practiced basketball showed significantly higher values of change for all tests assessed, compared to the control group. These findings are consistent with previous intervention studies in which basketball has been used as an intervention program in children. Significant improvements were found in speed, agility and muscle strength^36^, expressed in turn in better performance in throwing, jumping and running tasks^37,38^. Basketball practice typically involves repeated accelerations/decelerations, rapid changes of direction, and frequent jumping/landing actions, which map closely onto the capacities assessed here (10 × 5 m shuttle run, standing long jump, and six-minute walk), and may also contribute to upper-limb strength through repeated handling and stabilization demands (handgrip strength)^36–38^. These improvements may be related to the challenging characteristics of this sport in motor terms, training such competencies in an integrated manner with cognitive demands, driven by a constant need to adapt to the changing requirements of the game^62^.

In this study, phonological fluency was significantly associated with changes in long jump performance, thus evidencing a moderate negative correlation. This finding suggests that improvements in motor performance may be linked to variations in executive function, although the reverse direction of the relationship requires cautious interpretation. Complementarily, logistic regression analyses indicated that participants who improved their long jump were more likely to show better phonological fluency, although this trend did not reach the statistical significance level. Together, these results highlight a possible interaction between the motor and cognitive domains, but they also underscore the need to replicate these findings with larger sample sizes to clarify the directionality and strength of these associations.

### Strengths, limitations, and practical applications

One of the main strengths of this study lies in the implementation of a structured after-school basketball program in real-world school settings, which allowed us to evaluate its implementation in a naturalistic environment. The quasi-experimental design, with pre- and post-intervention measures and the inclusion of a control group, allowed us to observe greater pre-to-post improvements in both phonological fluency and physical fitness indicators, supporting the ecological validity of the findings. Furthermore, the integration of multiple assessment domains (executive function, physical fitness, anthropometric parameters, and physical activity level) provided a comprehensive view of the multifaceted associations of after-school basketball participation with child development outcomes. However, it is important to acknowledge some limitations. First, as no a priori sample size/power calculation was performed for this feasibility-oriented pilot, the modest sample size implies limited statistical power and an increased risk of type II error; thus, non-significant findings (e.g., semantic fluency) should not be interpreted as evidence of no effect. Consistent with this, sensitivity power estimates indicate that the present sample was primarily powered to detect large effects in the primary outcome; smaller effects may not have been detectable. Second, the absence of an active comparator limits causal inference: because the control group did not engage in a time-matched structured after-school activity, the observed between-group differences—particularly in phonological fluency and physical fitness—cannot be attributed specifically to basketball and may reflect nonspecific effects of increased physical activity and/or structured after-school engagement. Third, participants self-selected into the after-school basketball workshop, which may have introduced selection bias and residual confounding (e.g., higher motivation, parental support, or baseline activity preferences among enrolled children), further limiting basketball-specific causal inference. Fourth, the intervention period (12 weeks) may not have been long enough to show a significant impact on semantic fluency. Fifth, the use of a self-report questionnaire to measure physical activity (PAQ-C) may have introduced recall bias or social desirability. Finally, since this was a quasi-experimental design, it is possible that baseline differences were not completely controlled in the analyses. Additionally, baseline imbalances in several anthropometric and physical fitness variables may have influenced between-group comparisons; although we conducted baseline-adjusted ANCOVA analyses, residual confounding cannot be excluded given the non-randomized design and self-selection.

Despite these limitations, the study provides relevant practical applications. Participation in a structured after-school basketball program appears to be a feasible and promising strategy associated with improvements in phonological fluency and physical fitness in school-aged children. Given its accessibility and popularity, basketball can be incorporated in after-school programs as a low-cost, cognitively stimulating activity, which may support health and academic outcomes. It is recommended that future randomized controlled studies, with larger sample size and long-term follow-up, include active comparators to confirm these findings and further explore the mechanisms linking motor and cognitive development.

## Conclusions

This pilot study provides preliminary evidence that participation in a structured after-school basketball program may be associated with improvements in phonological fluency and physical fitness among school-aged children. However, because no active comparator was included, these findings should not be interpreted as basketball-specific effects. These findings are consistent with prior literature indicating that open-skill sports may foster cognitive engagement and executive function development. Accordingly, the results should be interpreted cautiously due to the exploratory nature of the study.

The small sample size, short intervention period, non-randomized group allocation (including voluntary self-selection into the basketball workshop), and the absence of an active comparator limit statistical power and generalizability and preclude basketball-specific causal inference. In addition, potential confounding variables—such as baseline differences and the use of self-reported measures of physical activity—should be acknowledged. Although baseline-adjusted ANCOVA sensitivity analyses were conducted, residual confounding cannot be excluded given the non-randomized design and self-selection.

Despite these limitations, the study supports the feasibility and acceptability of implementing cognitively enriched, sport-based interventions in authentic school environments. Basketball practice, as a dynamic and socially engaging activity, may represent a promising, low-cost approach to support physical and cognitive development in children. Future research employing longitudinal, multicenter, and randomized controlled designs with larger and more diverse samples is warranted to confirm these preliminary findings and elucidate the mechanisms linking open-skill sport participation to executive function enhancement.

## Data Availability

The datasets generated during this study are available from the corresponding author upon reasonable request.
